# The metabolic alterations within the normal appearing brain in patients with Hashimoto’s thyroiditis are correlated with hormonal changes

**DOI:** 10.1007/s11011-018-0318-z

**Published:** 2018-09-21

**Authors:** Joanna Bladowska, Marta Waliszewska-Prosół, Maria Ejma, Marek Sąsiadek

**Affiliations:** 1 Department of General Radiology, Interventional Radiology and Neuroradiology, Wrocław Medical University, Wrocław, Poland; 2 Department of Neurology, Wrocław Medical University, ul. Borowska 213, 50-556 Wrocław, Poland

**Keywords:** Hashimoto’s thyroiditis, Autoimmune thyroiditis, Cerebral metabolism, Magnetic resonance spectroscopy

## Abstract

Hashimoto’s thyroiditis (HT) is the most common autoimmune disease in humans usually associated with subsequent hypothyroidism. The purpose of the study was to assess metabolic alterations within the normal appearing brain in subjects with HT using MR spectroscopy (MRS) and to correlate MRS measurements with hormonal concentrations. Fifty-five HT patients (mean age 43.5 yrs) and 30 healthy controls (mean age 42.5 yrs) were examined with the use of a 1.5 T MR scanner. There were no signs of central nervous system involvement in the studied group. The MRS examinations were performed using the single voxel method. The voxels were placed in the left parietal white matter (PWM) and the posterior cingulate gyrus (PCG). The NAA/Cr, Cho/Cr, and mI/Cr ratios were calculated. The correlations between metabolite ratios and hormonal concentrations (TSH, fT3, fT4) as well as anti-TG and anti-TPO levels were also assessed. We found significantly (*p* < 0.05) decreased NAA/Cr ratios in PCG and PWM in HT subjects compared to the control group. There were no other significant differences in metabolite ratios. We observed significant positive correlations between the NAA/Cr ratio in PCG as well as the PWM and fT3 level. There was also a significant negative correlation between the Cho/Cr ratio in the PCG and fT4 level. MRS could be a sensitive biomarker capable of depicting early cerebral metabolic disturbances associated with HT. Our findings may indicate the reduction of neuronal activity within the normal appearing brain in patients with HT as well as suggesting that there is a possible biological association between thyroid dysfunction and cerebral metabolic changes.

## Introduction

Normal thyroid gland activity is essential for the optimal development, maturation, and function of the central nervous system (CNS). Neurological syndromes may develop despite the treatment of certain thyroid diseases. Hashimoto’s thyroiditis (HT) is the most common autoimmune disease in humans frequently leading to hypothyroidism (Ehlers and Schott 2014). People suffering from HT may have difficulty remembering things and problems with concentration. They may also experience excessive irritability, thought and sleep disorders, anxiety, mood swings and depression. They may be diagnosed with myopathy, neuropathy, ataxia, and encephalopathy. The exact cause of these symptoms has not yet fully been explained. Their pathomechanism is still being studied. It has been reported that among patients with hypothyroidism receiving biochemically adequate treatment, well-being and cognition performance remain reduced (Quinque et al. 2014; Wekking et al. 2005; Caturegli et al. 2014).

We hypothesized that since Hashimoto’s thyroiditis can impair functioning of the CNS, patients with this disease may present alterations in their cerebral metabolism, as measured by in vivo proton magnetic resonance spectroscopy (MRS). The aim of the study was to evaluate metabolic changes within the normal appearing brain in patients with HT using MR spectroscopy (MRS) and to correlate MRS measurements with clinical data.

## Materials and methods

### Patients

Fifty-five HT patients (50 females, 5 males) without central nervous system involvement (aged 25–64 years, mean age 43.5 yrs) and 30 age- and gender-matched healthy controls, (27 women and 3 men, mean age 42.5 yrs) were enrolled in the study. All the patients fulfilled the criteria for the diagnosis of Hashimoto’s thyroiditis (Caturegli et al. 2014). They underwent ultrasound examination of the thyroid gland and were evaluated by an endocrinologist. The duration of HT was 2–18 years (mean 45 months). The patients with HT were in the euthyreosis phase (normal TSH level), and were treated with levothyroxine.

The exclusion criteria were as follows:

1. Presence of any neurological disease (e.g., cerebrovascular disease, post-traumatic and post-toxic changes, inflammatory changes, multiple sclerosis, brain neoplasms);
2. Patients with other autoimmune diseases (e.g., CIDP, Sjögren Syndrome, systemic lupus erythematosus)
3. A history of psychiatric disease (e.g., depression, schizophrenia, ADHD, autism)
4. Patients with HT who were treated with medicines which change brain bioelectrical activity (e.g., steroids, neuroleptics, antiepileptic)
5. Patients with visual and hearing impairment and with symptoms of neurological deficit.

The neurological study protocol included a detailed neurological examination, with assessment of the patients’ mental state using the Mini-Mental State Examination (MMSE), the Clock Drawing Test (CDT) and the Symbol Digit Modalities Test (SDMT) to screen for cognitive impairment.

The study was conducted in accordance with the guidelines of the Wroclaw Medical University Ethics Committee for conducting research involving humans and approved by the Commission of Bioethics at Wroclaw Medical University (number of permission: KB-313/2013). Each patient had to provide their written consent to participate in the examination.

### The control group (CG)

The healthy control group consisted mainly of hospital staff from the Department of Radiology. The neurological CG protocol as well as the exclusion criteria were the same as in the study group. Thyroid and CNS diseases were excluded in the CG.

### MR imaging and MRS protocols

The MR examinations were conducted with the use of a 1.5 T MR scanner (Signa Hdx, GE Medical System) using a 16-channel coil dedicated for head and spine imaging. Before MRS examination conventional MR protocol for brain imaging was performed including axial, sagittal, and coronal T2-weighted images, axial T1-weighted images and FLAIR (fluid-attenuated inversion recovery sequences) images. The imaging protocol also comprised diffusion-weighted imaging (DWI). The main radiological inclusion criterion was the normal signal intensity of the gray and white matter without evidence of any cerebral atrophy on the MR examination.

The MRS studies were conducted by using the Single Voxel Spectroscopy (SVS) technique (PRESS sequence) as described in detail previously (Bladowska et al. 2014). The parameters of data acquisition were as follows: TR = 1500 ms, TE = 35 ms, number of acquisitions – 128, number of excitations – 8, time of acquisition – 3 min 45 s.

**Fig. 1:**
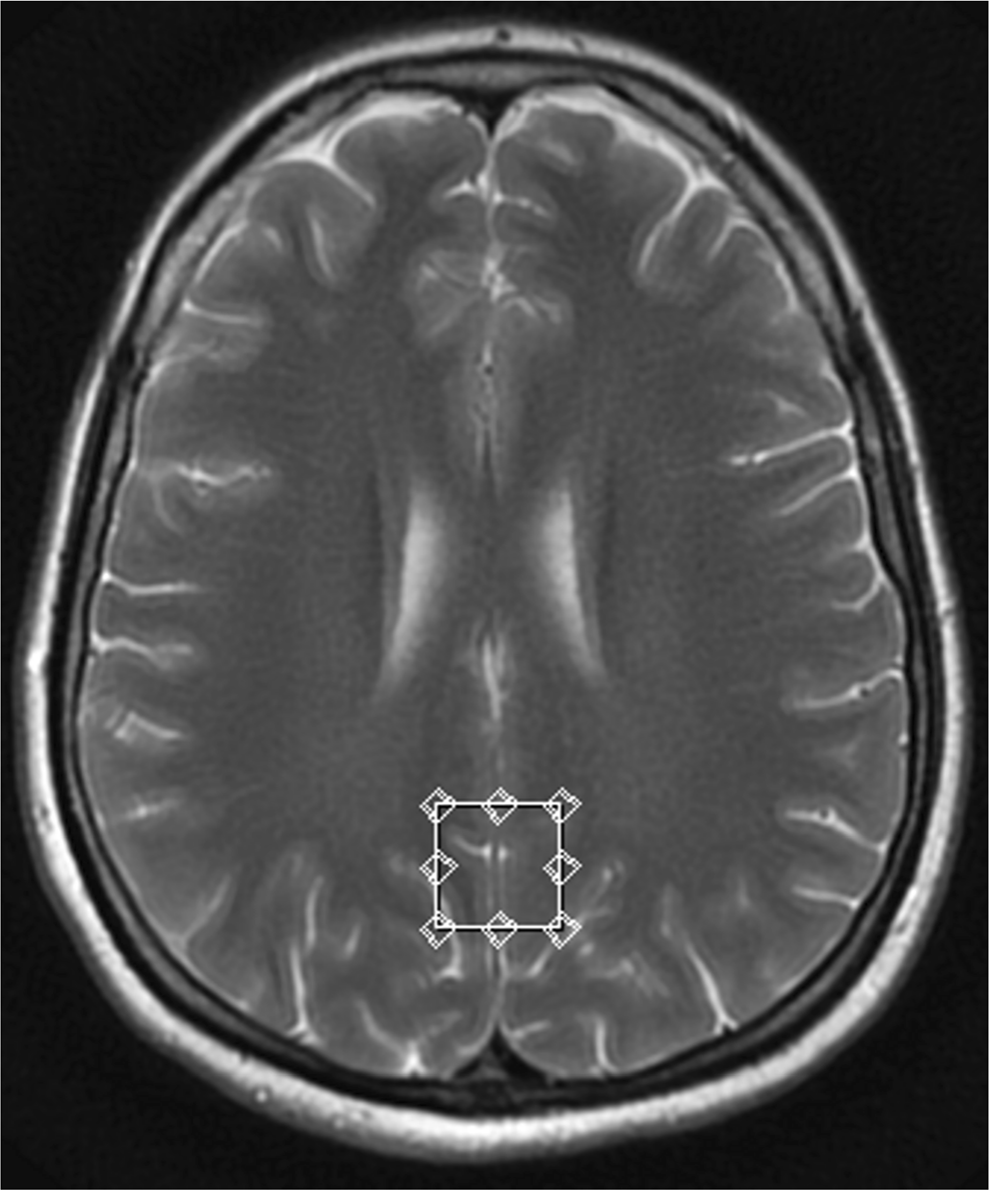
Single voxel magnetic resonance spectroscopy (MRS). Representative T2-weighted image (transverse cross-section) indicating voxel location in the posterior cingulate gyrus (PCG) region

**Fig. 2:**
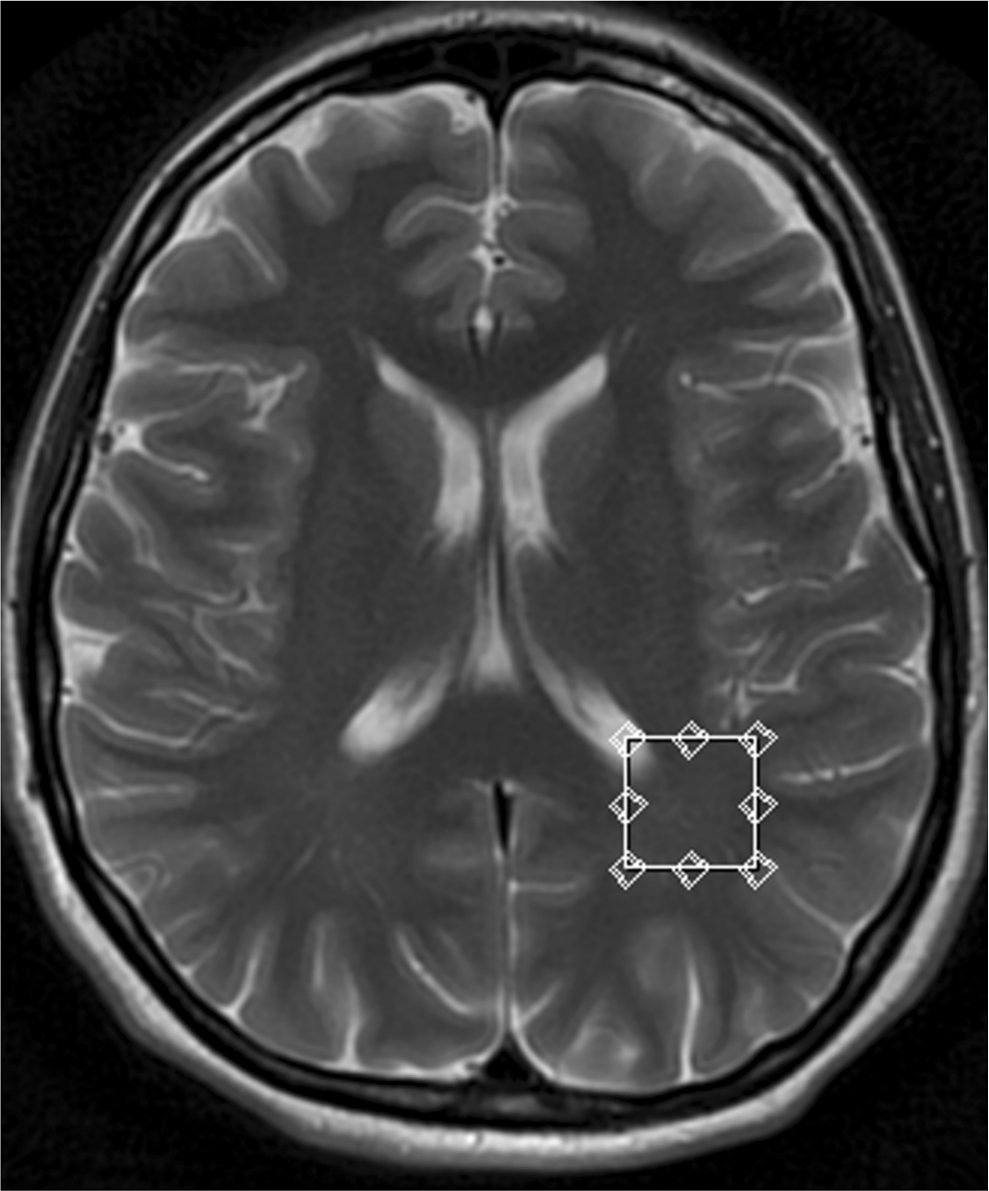
Single voxel magnetic resonance spectroscopy (MRS). Representative T2-weighted image (transverse cross-section) indicating voxel location in the left parietal (PWM) region

Using the localizing axial T2-weighted images of the brain, two voxels of 2x2x2cm (8cm^3^) were located in the gray and white matter. The voxels were placed in the following 2 regions: posterior cingulate gyrus - PCG (Fig. 1) and left parietal white matter – PWM (Fig. 2). The total acquisition time was 3 min 45 s for each voxel. Post-processing of MRS data was carried out on commercial workstations using algorithms provided by the manufacturer (GE workstation, ADW 4.4). Each spectrum was automatically fitted to four peaks corresponding to the levels of N-acetylaspartate (NAA) (2.02 ppm), total creatine (Cr) (3.03 ppm), choline-containing compounds (Cho) (3.23 ppm) and myo-inositol (mI) (3.56 ppm). Ratios of NAA, Cho and mI to creatine (NAA/Cr, Cho/Cr, mI/Cr, respectively) were calculated and analyzed.

Additionally, the correlations between metabolite ratios and hormonal concentrations (TSH, fT3, fT4) as well as anti-TG and anti-TPO levels were also assessed.

### Statistical analysis

The MRS measurements in patients with HT and healthy subjects were compared using the Student T-test as well as using ANOVA analysis in order to compare patients with HT to the control group according to the length of the disease.

Possible sex influence between the study and control group were analyzed using the chi^2^ test.

Additionally, in order to assess sensitivity, specificity and accuracy of MRS in distinguishing HT patients and normal controls, the receiver-operating characteristic (ROC) analysis was performed for metabolite ratios showing the most significant differences between these two groups. The rate of accuracy was based on the area under the ROC curve.

Associations between hormonal concentrations and MRS measurements in patients with Hashimoto’s thyroiditis were assessed using Pearson’s correlation coefficient.

For all tests, a significance level of 0.05 was chosen. The STATISTICA software package was used.

## Results

There were no significant differences in age (analysis of variance; *p* = 0.1) and sex distribution (chi^2^ = 0.6, *p* = 0.82) between HT patients and control group.

### Neurological examination, cognition and thyroid function

The neurological examination was normal in 48 patients (87.3%). In 7 cases (12.7%) there were modest symptoms of peripheral nervous system (PNS) involvement, such as attenuation of superficial sensation in the distal parts of the legs, as well as decreased knee and ankle reflexes.

In all HT patients the results of the MMSE, CDT, and SDMT were normal.

The mean value for TSH was 1.87 ± 1.24 UIU/ml (normal value 0.35–5.6 UIU/ml), fT3 2.92 ± 0.54 pg/ml (normal value 2.5–3.9 pg/ml), fT4 1.07 ± 0.27 ng/dl (normal value 0.61–1.12 ng/dl), anti-TG 107.93 ± 230.47 IU/ml anti-TPO 455.3 ± 369.24 IU/ml.

### Comparison of MRS measurements between HT patients and control group (CG)

Table 1 shows the mean values and standard deviations of the metabolite/creatine ratios in each brain region in patients with HT and normal control subjects.

**Table 1:** Mean values and standard deviations (SD) of the metabolite/creatine ratios in PCG and PWM regions with the results of the student t-test

Location	NAA/Cr Mean values (SD)	Cho/Cr Mean values (SD)	mI/Cr Mean values (SD)
PCG (Posterior cingulate gyrus)			
Control group	1.77 (0.06)	0.58 (0.06)	0.62(0.05)
HT patients	1.65 (0.12)	0.58 (0.06)	0.61 (0.10)
	*p* = 0.003	*p* = 0.864	*p* = 0.866
PWM (Parietal white matter)			
Control group	1.97(0.09)	1.09(0.13)	0.62(0.10)
HT patients	1.88 (0.14)	1.08 (0.14)	0.65 (0.08)
	*p* = 0.036	*p* = 0.715	*p* = 0.276

Compared to the control group (CG), subjects with HT revealed a statistically significant decrease of the NAA/Cr ratios in both PCG (*p* = 0.000000002) and PWM (*p* = 0.000000005) regions.

Other metabolite ratios in all the analyzed regions showed no statistically significant differences between the evaluated groups (Table 1).

**Fig. 3:**
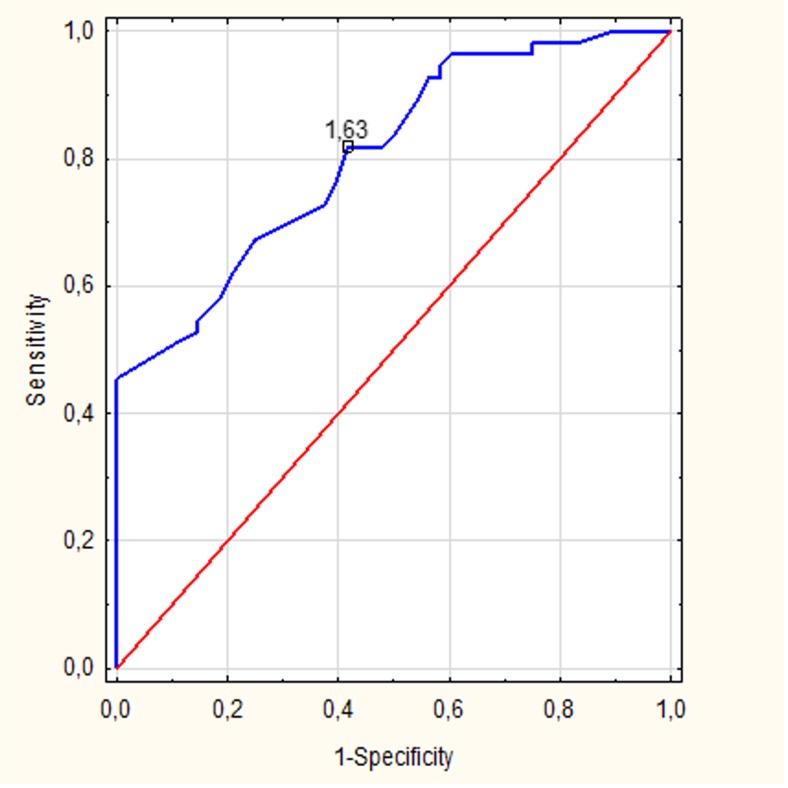
The receiver-operating characteristic (ROC) curves for the N-acetylaspartate/Creatine (NAA/Cr) ratios from the posterior cingulate gyrus (PCG) area

**Fig. 4:**
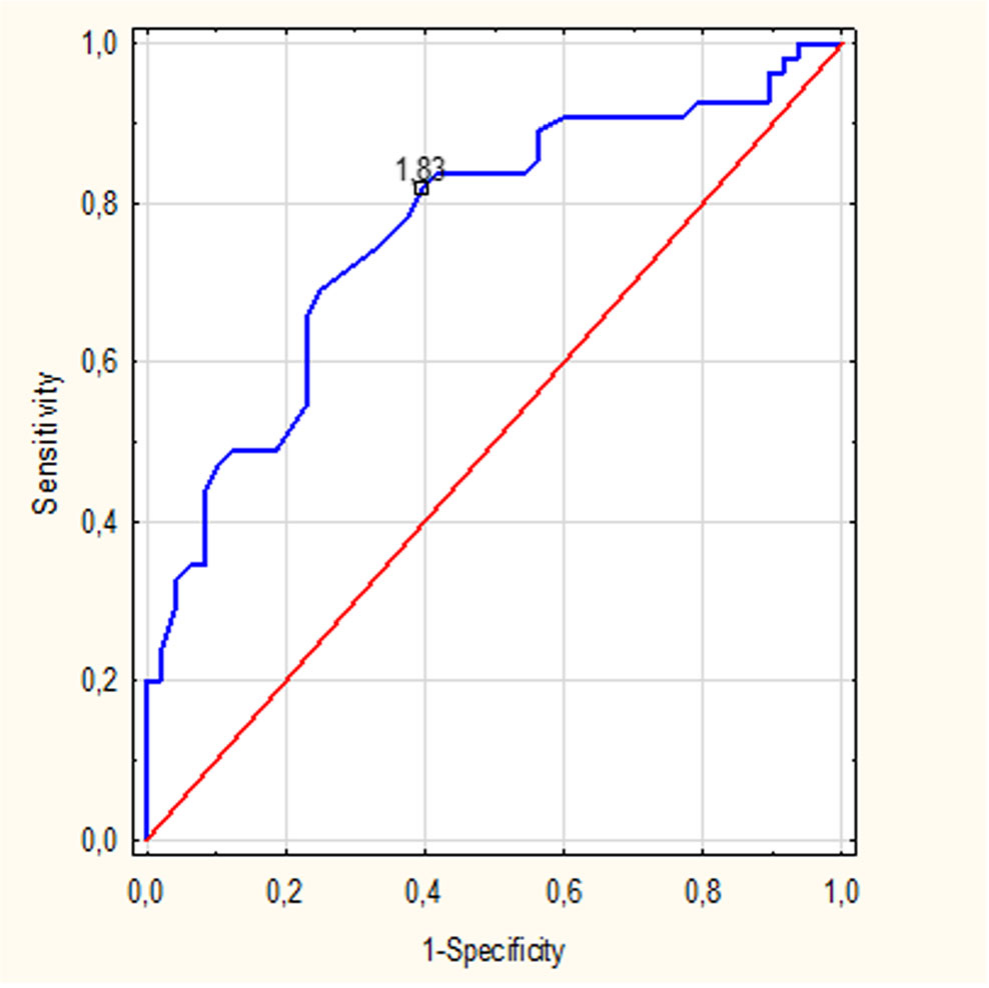
The receiver-operating characteristic (ROC) curves for the N-acetylaspartate/Creatine (NAA/Cr) ratios from the parietal white matter (PWM) region

ROC analysis was performed separately for the PCG and PWM region using NAA/Cr ratios. Both ROC curves demonstrated good diagnostic accuracy with the area under the curve 0.79 for the PCG region (Fig. 3) and 0.72 for the PWM region (Fig. 4). When a cut off value for PCG NAA/Cr ratio was fixed at <1.63, the sensitivity and specificity of MRS in distinguishing HT patients and normal controls was 81 and 58%, respectively. When a cut off value for PWM NAA/Cr ratio was fixed at <1.83, the sensitivity and specificity reached 81 and 60%, respectively (Table 2).

**Table 2:** Results of ROC analyses for the NAA/Cr ratios in PCG and PWM regions

	Cut point values	Sensitivity	Specificity	Accuracy
NAA/Cr PCG	< 1.63	0.81	0.58	0.79
NAA/Cr PWM	< 1.83	0.81	0.6	0.72

### Comparison of MRS measurements between HT patients and control group (CG) according to the length of the disease

In order to compare HT patients to CG according to the duration of the disease, HT patients were divided in four group as follows: group 1 (duration of the disease 2–5 years) – 12 subjects; group 2 (duration of the disease 6–9 years) – 15 subjects; group 3 (duration of the disease 10–13 years) – 18 subjects; group 4 (duration of the disease 14–18 years) – 10 subjects.

Compared to CG, subjects with HT showed a statistically significant decrease of the NAA/Cr ratios in each group in the PCG area as well as in group 2, 3 and 4 in the PWM region (Table 3). There was no statistically significant difference between HT patients in group 1 and CG in PWM, although the NAA/Cr ratio in group 1 was lower compared to CG. The results also revealed that the longer the HT disease the lower the NAA/Cr ratio in both PCG as well as PWM regions.

**Table 3:** Mean values and standard deviations (SD) of the NAA/Cr ratios in PCG and PWM regions with the results depending on the duration of the disease

Duration of the disease	Group 1 2–5 y	Group 2 6–9 y	Group 3 10–13 y	Group 4 14–18 y
Number of patients	12	15	18	10
PCG (Posterior cingulate gyrus)				
Control group	1.77 (0.09)	1.77 (0.09)	1.77 (0.09)	1.77 (0.09)
HT patients	1.65 (0.09)	1.61 (0.08)	1.50 (0,11)	1.45 (0.09)
	*p* = 0.023	*p* = 0.01	*p* = 0.009	*p* = 0.001
PWM (Parietal white matter)				
Control group	1.97 (0.13)	1.97 (0.13)	1.97 (0.13)	1.97 (0.13)
HT patients	1.81 (0.13)	1.76 (0.14)	1.70 (0.14)	1.61 (0.12)
	p = 0.05	*p* = 0.028	p = 0.01	*p* = 0.002

### Correlations of MRS measurements with hormonal concentrations in patients with HT

**Fig. 5:**
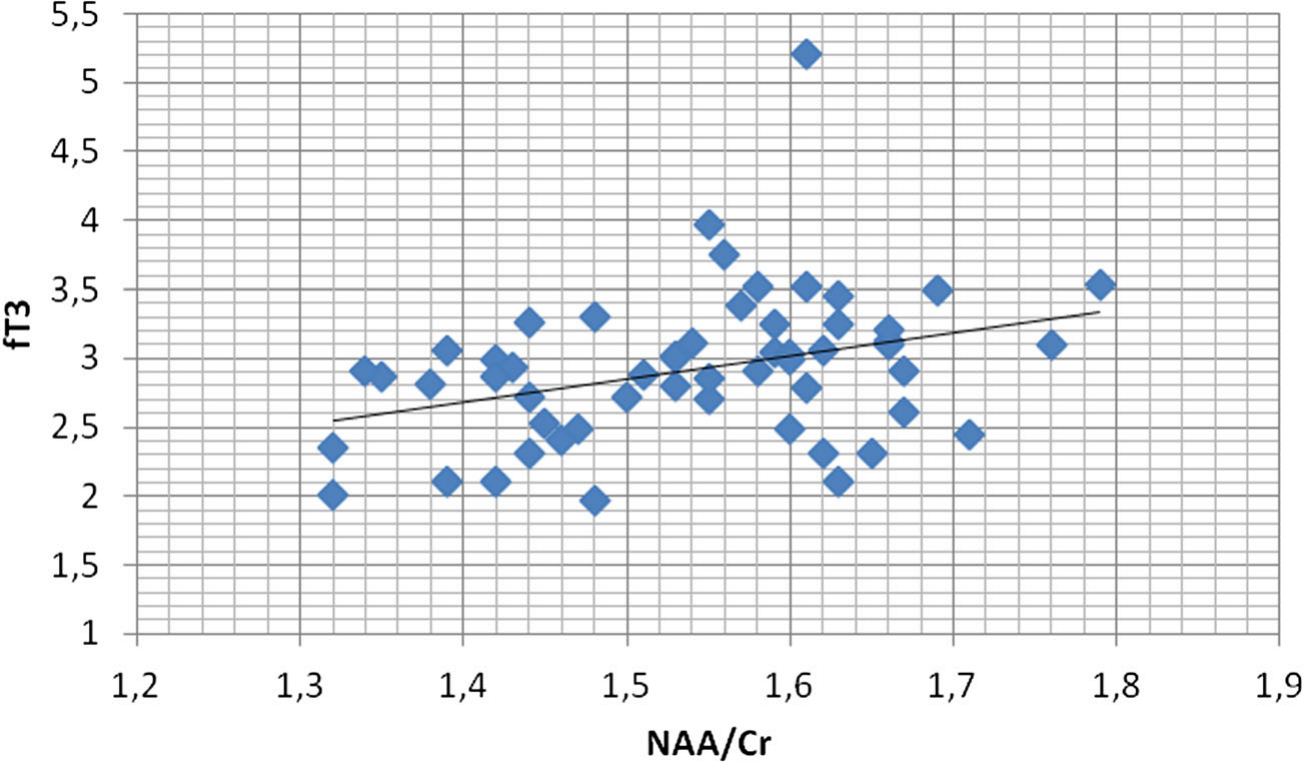
Correlation of PCG NAA/Cr ratios with fT3 concentrations. In patients with Hashimoto’s disease there was a significant positive correlation between N-acetylaspartate/Creatine (NAA/Cr) ratio in the posterior cingulate gyrus (PCG) region and fT3 concentration (*r* = 0.344, *p* < 0.05)

**Fig. 6:**
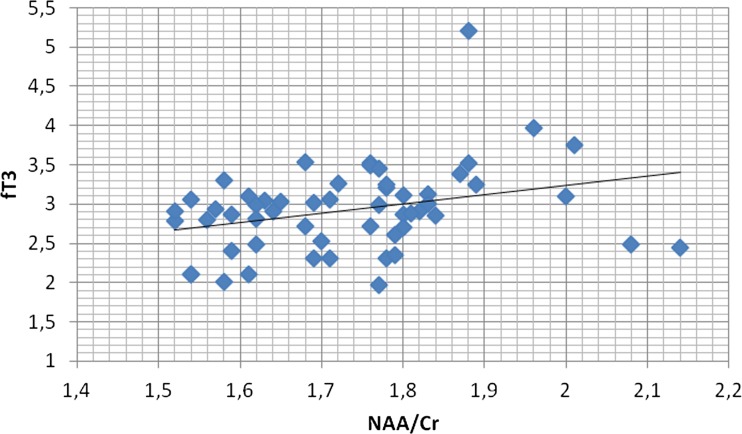
Correlation of PWM NAA/Cr ratios with fT3 concentrations. In patients with Hashimoto’s disease there was a significant positive correlation between N-acetylaspartate/Creatine (NAA/Cr) ratio in the parietal white matter (PWM) region and fT3 concentration (*r* = 0.311, p < 0.05)

NAA/Cr ratios in the PCG region as well as in the PWM area showed significant positive correlations with fT3 concentrations (r = 0.344, *p* < 0.05; r = 0.311, *p* < 0.05, respectively) (Figs. 5 and 6).

**Fig. 7:**
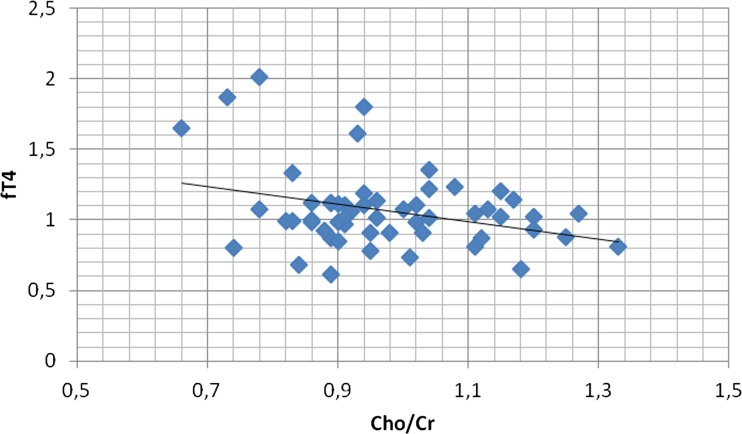
Correlation of PWM Cho/Cr ratios with fT4 concentrations. In patients with Hashimoto’s disease there was a significant negative correlation between Choline/Creatine (Cho/Cr) ratio in the parietal white matter (PWM) region and fT4 concentration (*r* = − 0.322, p < 0.05)

There was also a significant negative correlation between the Cho/Cr ratio in PWM and fT4 level (*r* = − 0.322, p < 0.05) (Fig. 7).

### Correlations of MRS measurements with anti-TG and anti-TPO levels in patients with HT

There were no statistically significant correlations between NAA/Cr, Cho/Cr, and mI/Cr ratios in the evaluated brain regions, and anti-TG as well as anti-TPO levels.

## Discussion

It has been reported that patients with adequately treated Hashimoto’s thyroiditis have an increased risk of depression and anxiety (Ott et al. 2011). In the available literature there are only a few brain imaging studies on subjects with HT, including three SPECT studies showing diffuse hypoperfusion (Zettinig et al. 2003; Piga et al. 2004; Kaya et al. 2007), a single voxel-based morphometry study (Leyhe et al. 2013) as well as a report on fMRI findings in patients with HT (Quinque et al. 2014). The results of these papers suggested brain alterations in patients with HT, however findings on other brain imaging methods are still missing. To the best of our knowledge, this study showing metabolic changes in patients with HT using MRS examinations is the first report in world literature.

All the subjects of our HT patients had compensated thyroid function as well as a normal neurological and mental state.

The aim of our study was to assess the metabolic alterations in subjects with HT within normal appearing white and gray matter in MRS and to correlate MRS measurements with hormonal concentrations.

MRS has enabled the in vivo assessment of certain metabolites in a variety of pathologic processes that affect the CNS. MRS can show the changes in metabolite profiles in normal appearing white matter (NAWM) and normal appearing gray matter (NAGM) (Mandal 2012; Bladowska et al. 2013a).

We found a significant decrease of NAA/Cr ratio in the posterior cingulate regions (PCG) and in the white matter of the left parietal lobe (PWM). The reduction of NAA/Cr ratios may suggest loss of neuronal activity within normal appearing gray and white matter in patients with Hashimoto’s thyroiditis.

Moreover, assessment of the NAA/Cr ratios within gray and white matter turned out to be a useful tool enabling the distinguishing of HT patients from healthy controls reaching high sensitivity and specificity rates (81 and 58% for the PCG region and 81 and 60% for the PWM area, respectively). We also found that measurements of the NAA/Cr ratios showed high accuracy in distinguishing HT patients from controls (0.79 for the PCG region and 0.72 for the PWM area, respectively).

Our results revealed an interesting relationship according to the duration of the disease indicating that the longer the disease the lower the NAA/Cr ratio in HT patients compared to healthy people. There was a statistically significant decrease of the NAA/Cr ratios in HT patients in groups with different disease length in comparison to the healthy controls, except from the group 1 (duration of the disease 2–5 years) in the PWM region, however the *p* value was close to the margin of statistical significance (*p* = 0.05).

Furthermore, we observed significant positive correlations between NAA/Cr ratios in the PCG region as well as in the PWM area and fT3 concentrations, which means that decreased NAA/Cr ratios were significantly associated with lower fT3 concentrations.

The parietal region and posterior cingulate gyrus were intentionally chosen for analysis as these areas have been considered to best correspond with cognitive function in MRS studies in healthy persons as well as patients with mild cognitive impairment and dementia. The PCG area as a part of the limbic system has been found to show mutual connections with the hippocampus and medial temporal lobe. The posterior cingulate gyrus region and precuneus is called a thalamocortical portion of Papez’ circuit because of the functional connectivity with the thalamus. This part of the brain is engaged in memory and learning processes which are impaired in the course of dementia syndromes (Bladowska et al. 2014, 2013b; Nesteruk et al. 2016). Several studies have reported that the posterior cingulate area could be the most severely involved region in dementia and predementia patients. Metabolic changes in this area are believed to be predictors of cognitive decline in presymptomatic subjects, suggesting that the pathologic process begins well before even mild dementia is diagnosed clinically (Bladowska et al. 2014, 2013b; Zimny et al. 2011).

Our results of the reduction of NAA/Cr ratios in PCG as well as the positive correlations between NAA/Cr ratios in the PCG region and fT3 concentrations may suggest that these patients are prone to cognitive decline.

On the other hand, we found a significant negative correlation between the Cho/Cr ratio in PWM and fT4 level. Choline is a marker of cellular membrane turnover. Elevated levels of Cho are believed to reflect cellular proliferation due to infection or inflammation (Bladowska et al. 2013a). Our results showed that increased Cho/Cr ratios were significantly associated with lower fT3 levels. These findings suggest that there may be a biological link between hypothyroidism and increased cellular membrane turnover in the white matter, perhaps due to some inflammatory process ongoing in the brain, which may underlie neuronal dysfunction in subjects with HD. Furthermore, it seems that the inflammation process may play a role in the pathogenesis of brain alterations in patients with HT. Our results are consistent with the data from the literature indicating potential mechanisms which can contribute to brain disequilibrium in Hashimoto’s thyroiditis (Leyhe and Müssig 2014). One of the possible mechanisms involved in brain injury in the course of HT involves cytokines (Leyhe and Müssig 2014). In previous studies an increase has been reported in the production of monocyte cytokine, comprising monocyte chemoattractant protein-1 (MCP-1), and T lymphocyte cytokine production, including interferon gamma (INF-γ) and tumor necrosis factor alpha (TNF-α) in patients with Hashimoto’s thyroiditis (Leyhe and Müssig 2014; Kokkotou et al. 2002; Karanikas et al. 2005). Inflammatory cytokines seem to have a negative influence on multiple neurotransmitters, including dopamine, serotonin, and glutamate, by changing processes of their synthesis, release, as well as reuptake. These undesirable effects cause some modifications in various brain cycles and subsequently significant disturbances in motor activity and motivation but also they can affect processes of arousal and alarm as well as anxiety (Leyhe and Müssig 2014; Miller et al. 2013).

## Conclusions

In our opinion, MRS could be a sensitive marker of early cerebral metabolic disturbances associated with Hashimoto’s thyroiditis. Moreover, our findings suggest that there is a biological link between thyroid dysfunction and cerebral metabolic changes as measured by in vivo proton magnetic resonance spectroscopy. To the best of our knowledge, the results of MRS measurements in the normal appearing brain in patients with Hashimoto’s thyroiditis have never been published before in world literature.
